# The Role of Selenitetriglycerides in Enhancing Antioxidant Defense Mechanisms in Peripartum Holstein-Friesian Cows

**DOI:** 10.3390/ani14040610

**Published:** 2024-02-13

**Authors:** Katarzyna Żarczyńska, Paweł Brym, Dawid Tobolski

**Affiliations:** 1 Department and Clinic of Internal Diseases, Faculty of Veterinary Medicine, University of Warmia and Mazury in Olsztyn, 10-719 Olsztyn, Poland; katarzyna.zarczynska@uwm.edu.pl; 2 Department of Animal Genetics, Faculty of Animal Bioengineering, University of Warmia and Mazury in Olsztyn, 10-719 Olsztyn, Poland; pawbrym@uwm.edu.pl; 3 Institute of Animal Reproduction and Food Research, Polish Academy of Sciences, 10-747 Olsztyn, Poland

**Keywords:** selenitetriglycerides, antioxidants, gene expression, Holstein-Friesian cows, peripartum period, selenium supplementation

## Abstract

**Simple Summary:**

The transition period for high-yielding dairy cows, marked by calving and the onset of lactation, presents a substantial risk of oxidative stress. This stress can have adverse effects on cow health and milk production yield. Our research aimed to assess the effectiveness of selenitetriglycerides as an oral supplement for alleviating oxidative stress in periparturient cows. We conducted a trial involving 12 dairy cows, divided into two groups: one group received the selenitetriglyceride supplement, while the other served as a control group receiving placebo supplementation. The study assessed key indicators of oxidative stress and overall health, including serum selenium levels, non-esterified fatty acids, glutathione peroxidase, catalase concentrations, and liver gene expression profiles related to antioxidant activity. The results demonstrated a significant enhancement in antioxidant levels and the stabilization of oxidative markers in supplemented cows. These findings suggest that selenitetriglyceride supplementation may serve as an effective strategy to enhance antioxidant defenses in dairy cows during this critical period. The implications of this study extend to improving both animal welfare and productivity in the dairy industry, potentially benefiting the agricultural sector and consumer health by promoting more sustainable dairy practices.

**Abstract:**

The transition period in high-yielding dairy cows is a critical phase marked by an elevated risk of oxidative stress. This study evaluated the effect of oral selenitetriglyceride supplementation on oxidative stress management in periparturient cows. A controlled experiment was conducted on 12 cows, divided into two groups: the experimental group (STG) received selenitetriglycerides (0.5 mg Se/kg BW), while the control group (CON) was given a placebo, starting 12 days before calving until the calving day. Blood and liver tissue samples were collected at predetermined intervals around the time of parturition. The study observed a significant increase in serum selenium levels and NEFA stabilization in the STG group compared with the control. Antioxidant parameters indicated elevated GSH-Px and CAT concentrations in the STG group. Liver gene expression analysis revealed a significant increase in SOD2 mRNA levels in the STG group (FC = 4.68, *p* < 0.01). Conversely, GSH-Px3 expression significantly decreased (FC = 0.10, *p* < 0.05) on the 7th day postpartum in the CON group. However, SOD1, SOD3, and CAT expressions remained stable in both groups. These findings highlight the beneficial role of selenitetriglycerides in enhancing antioxidant capacity and influencing specific gene expressions associated with oxidative stress management in dairy cows during the peripartum period.

## 1. Introduction

Intensive biochemical processes occurring in the bodies of high-yielding dairy cows result in a significant increase in oxygen demand. The consequence of these transformations is the production of significant amounts of free oxygen radicals (FOR) and other reactive oxygen species (ROS). These compounds are formed spontaneously as by-products but are also indispensable products of cellular metabolism. A small concentration of reactive oxygen species is harmless to the body and even necessary in certain situations. Phagocytic cells use free radicals to eliminate pathogens [1]. Under homeostasis conditions, reactive oxygen species are constantly inactivated by antioxidants of both endo- and exogenous origin [2]. An increase in the production of free radicals, a decrease in the amount of antioxidants, and a decrease in the activity of enzymatic systems responsible for the removal of ROS mean that they are not effectively eliminated from the body. The balance is disturbed, and pro-oxidant processes outweigh antioxidant processes, which is called oxidative stress. During the transition period, high-yielding dairy cows are significantly exposed to the effects of free radicals. Their excessive production is mainly caused by heat stress, high milk production, and an incorrectly balanced diet, especially in terms of the supply of minerals and vitamins [1,3]. Oxidative stress may lead to the occurrence of a number of diseases of the perinatal period and early lactation, including metabolic diseases, but also infections related to the dysfunction of the immune system [4].

The harmful effects of reactive oxygen species in organisms are counteracted by antioxidants located in cellular defense systems. This group of compounds includes any substance that delays, prevents, or eliminates the effects of oxidation [5]. These include non-enzymatic and enzymatic factors. A non-enzymatic system is composed of substances that donate their electrons to free radicals, i.e., they transform into an oxidized form characterized by low reactivity, thus preventing the oxidation of other components. This group of compounds, which are commonly called free radical scavengers, includes coenzyme Q10, flavonoids, reduced glutathione (GSH), vitamins A, C, E, and β-carotene. The enzymatic system consists of specialized enzymes responsible for carrying out reactions that remove free radicals and prevent their formation. The main enzymatic antioxidants are superoxide dismutase (SOD), which carries out the dismutation reaction of superoxide anion to hydrogen peroxide; catalase (CAT), which decomposes H_2_O_2_ to water and oxygen; and glutathione peroxidases (GSH-Px), which reduce H_2_O_2_ or lipid peroxides with the simultaneous oxidation of glutathione [6]. The following microelements are necessary for the proper functioning of the enzymatic system: selenium (Se), copper (Cu), zinc (Zn), iron (Fe), iodine (I), and manganese (Mn), because they are the active centers of these enzymes [7].

Selenium (Se) is considered a microelement of fundamental importance for human and animal health. This element is characterized by strong antioxidant, anticancer, and antiviral properties and stimulates the functioning of the immune system [8]. Glutathione peroxidases are a family of antioxidant enzymes with selenocysteine present in their active sites, which constitute an important group of seleno-dependent proteins in mammals [9]. GSH-Px has a greater affinity for H_2_O_2_ than catalase and can neutralize it at much lower concentrations. The GSH-Px family is represented in mammals by eight types of this protein (GSH-Px1 through GSH-Px8). In mammals, GSH-Px1-GSH-Px4 and GSH-Px6 are considered selenium-containing enzymes that have antioxidant functions, while GSH-Px6 is expressed only in the human body [10,11]. Moreover, apart from GSH-Px, many other genes related to the antioxidant properties of selenium have been identified: selenoprotein P (SEPP1), selenoprotein 15 (SEP15), thioredoxin reductase 1 and 2 (TRXNRD1,2), peroxiredoxin 1–6 (PRDX1–6), selenium binding protein 1 (SELENBP1), superoxide dismutase 1, 2, 3 (SOD1,2,3), and catalase (CAT) [12,13]. Reducing the level of selenium and other antioxidants in the diet causes a decrease in SOD activity, which increases the level of free radicals in the body. It is suggested that this may be influenced by GSH-Px4, which is most likely involved in the activation of SOD [14]. This complex network of selenium-dependent antioxidant pathways highlights the key role of this element in limiting the occurrence of oxidative stress, emphasizing the need for adequate selenium intake in both human and animal diets.

In order to supplement Se deficiencies, inorganic (selenite, selenate) and organic (selenomethionine, dimethylselenide) forms of selenium are traditionally used. Due to the increased biological use of selenium in the body, selenium is also used in the form of selenized yeasts, selenium bound to Chlorella algae biomass, and nanoforms of this element [13]. Selenitetriglycerides are a new form of organic selenium in which Se is in the 4th oxidation state. These compounds are created as a result of the chemical modification of sunflower oil with selenic acid. Due to their lipophilic properties, they are very well absorbed after oral, subcutaneous, and intraperitoneal administration. Selenitetriglycerides are characterized by relatively low toxicity. Studies on rats have shown that the average lethal dose of selenitetriglycerides in the form of 2% and 5% solutions is 100 and 68 mg/kg body weight (BW), respectively [15]. For comparison, the lethal dose of selenium administered in the form of sodium selenate ranges from 1 to 5 mg/kg BW [16]. The effectiveness and safety of oral administration of selenitetriglycerides have also been demonstrated in cows [17], camels [18], calves [19], and pigs [20]. As previous studies conducted on mice [21], rats [22], calves [19], and pigs [20] have shown, this form of selenium significantly affects the activation of selenium-dependent antioxidants after both single and long-term administration of selenitetriglycerides. This finding underscores the potential of selenitetriglycerides as a superior form of selenium supplementation, offering a promising approach for more effective management of selenium deficiency in a variety of animal species.

There is no information in the available literature on the impact of the use of selenitetriglycerides on the body of cows during the most critical period of their physiological cycle, which is the transition period. Therefore, the aim of this study was to investigate changes in peripheral blood metabolites, antioxidant indices, and the expression of liver genes involved in antioxidant processes in Holstein-Friesian cows during the peripartum period, after oral supplementation with selenitetriglycerides and without such supplementation.

## 2. Materials and Methods

### 2.1. Animals and Group Assignment

The study was conducted on 12 Holstein-Friesian cows from a single farm in northeastern Poland, divided into two equal groups. The cows constituted a homogeneous group in terms of nutrition (Table 1 and Table 2), age (third pregnancy), and BCS condition (3.5–3.75). The material was collected between January and April in order to exclude the impact of heat stress on the cows.

The first group—experimental (STG)—consists of cows that received daily oral selenitetriglycerides at a dose of 0.5 mg Se/kg BW, starting from the 12th day before the expected date of calving until the day of delivery. The second group—control (CON)—included cows that received 12 mL/animal of sunflower oil at the same time.

### 2.2. Blood Sampling, Biochemical Components, and Antioxidant Analyses

Blood samples were collected from all cows five times: on the 12th and 3rd days before calving and on the 1st, 4th, and 7th days after calving. The samples were taken from the median caudal vein approximately two hours after the morning feeding, using a vacuum system for venous blood collection consisting of a system needle (20G 0.9 × 38 mm) and a holder (BD Vacutainer, Becton Dickinson, Franklin Lakes, NJ, USA). To obtain serum, blood was collected into tubes with clotting activator (9 mL, Vacuette, Greiner Bio-One, Kremsmünster, Austria), while whole blood was collected in K_2_EDTA tubes (4 mL, Vacuette, Greiner Bio-One, Kremsmünster, Austria). The blood was centrifuged for 15 min at 1500 rpm. After centrifugation, the obtained blood cells were placed in a freezer at −80 °C and stored until analysis. Biochemical parameters were determined in the obtained serum, and then the rest of the material was placed in a freezer and stored at −80 °C to determine the selenium concentration. Serum Se concentration was determined by hydride generation-flame atomic absorption spectrometry (Unicam 939 Solar Spectrophotometer, Cambridge, UK). The following biochemical parameters were determined: activity of aspartate aminotransferase (AST), gamma-glutamyl transferase (GGT), and the concentrations of glucose (GLU), triglycerides (TG), cholesterol (CHOL), non-esterified fatty acids (NEFA), β-hydroxybutyrate (BHB), total protein (TP), and albumin (ALB) (Cormay ACCENT 200 Automatic Biochemical Analyzer and Cormay diagnostic kits, Poland). Concentrations of glutathione peroxidase (GSH-Px), superoxide dismutase (SOD), and catalase (CAT) were determined with Microplate Scanning Spectrophotometer Bio Tek Power Wave XS with the Bovine ELISA Kit (Bioassay Technology Laboratory, Szanghaj, China).

### 2.3. Liver Biopsy and RNA Extraction

**Figure 1:**
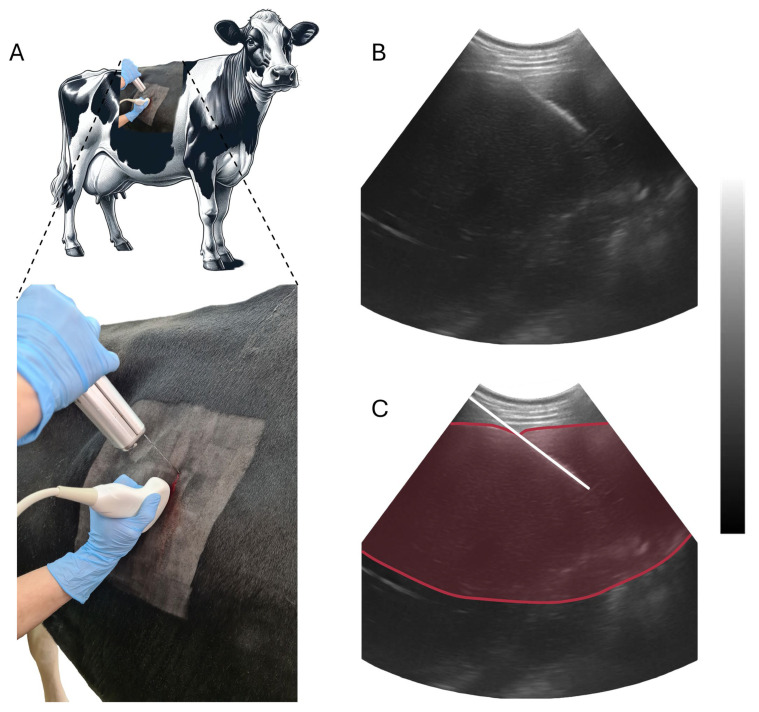
Schematic representation of the liver biopsy procedure performed. Panel (**A**) depicts the anatomical site for biopsy on the cow and the insertion of the biopsy needle, guided by ultrasound. Panel (**B**) shows an ultrasound image capturing a cross-sectional view of the bovine liver during the biopsy. Panel (**C**) provides an enhanced ultrasound image highlighting the needle trajectory (white line) with a red-delineated liver area. The procedure utilized a convex probe (5 MHz) and an ultrasound machine (4 Vet Slim, Dramiński, Olsztyn, Poland) for navigation and a sterile, percutaneous needle biopsy instrument (Pro-Mag Ultra, Argon Medical Devices, Plano, TX, USA) for tissue sampling.

The liver tissue was biopsied from all cows from intercostal space 9–11 under ultrasound guidance (convex probe—5 MHz, ultrasound—4 Vet Slim, Dramiński, Olsztyn, Poland) by a skilled veterinarian in 24 h postpartum and 7 days postpartum. A sterile, percutaneous needle biopsy (diameter 1.6 mm and length 20 cm, Pro-Mag Ultra, Argon Medical Devices, Plano, TX, USA) and an automatic biopsy instrument (Pro-Mag^TM^ Ultra, Argon Medical Devices, Plano, TX, USA) were used to collect tissue samples from each cow twice during each sampling session. The procedure was performed under local anesthesia (Polocainum Hydrochloricum 5% cum Adrenalino 0.005%, Biowet-Drwalew, Drwalew, Poland) within 2 h after the morning feeding. The tissue samples were stored in Eppendorf Safe-Lock Tubes (Eppendorf SE, Hamburg, Germany), which are sterile and low-temperature-resistant. They were then transported in liquid nitrogen and stored at −80 °C until analysis. The procedure for performing a liver biopsy under ultrasound guidance is depicted in Figure 1.

Liver biopsy samples were homogenized using 5 mm stainless steel beads in the TissueLyser LT system (Qiagen, Hilden, Germany), and total RNA was extracted using the Total RNA Mini Plus Kit (A&A Biotechnology, Gdynia, Poland) according to the manufacturer’s protocol. The resulting RNA preparations were digested with DNase, purified, and concentrated using the Clean-Up RNA Concentrator Kit (A&A Biotechnology, Gdynia, Poland), also according to the manufacturer’s instructions. RNA concentration, purity, and integrity were determined by measurements using a NanoDrop ND1000 spectrophotometer (Thermo Scientific, Waltham, MA, USA) and an Agilent 2100 Bioanalyzer with Agilent RNA 6000 Nano Kit (Agilent Technologies, Santa Clara, CA, USA). RNA samples were stored at −80 °C until cDNA synthesis.

### 2.4. cDNA Synthesis and RT-qPCR Analysis

1000 ng of oligo(dT)_18_ primed total RNA was reverse transcribed using the Maxima First Strand cDNA Synthesis Kit with dsDNase according to the manufacturer’s instructions (ThermoScientific, Waltham, MA, USA). The final cDNA products were diluted 100-fold prior to use in RT-qPCR, aliquoted, and stored at −20 °C. Real-time PCR reactions were performed in 96-well plate format on a LightCycler^®^ LC 480 II (Roche, Basel, Switzerland) using primers listed (Table 3) and the LightCycler 480 SYBR Green I Master Reagent Kit (Roche, Basel, Switzerland) according to the manufacturer’s instructions. The cycling conditions were as follows: pre-incubation at 95 °C for 5 min followed by 45 cycles, with each cycle including 95 °C for 10 s, annealing temperature listed (Table 3) for 10 s, and extension at 72 °C for 10 s. Then a melting curve was produced to confirm a single gene-specific peak and to detect primer–dimer formation by a rise of temperature to 95 °C for 5 s, cooling to 65 °C for 1 min, and stepwise increasing of temperature ranging from 65 to 97 °C at ramp rate of 0.11 °C/s with continuous monitoring of the fluorescence. For RT-qPCR data normalization, a pair of GAPDH and RPL32 reference genes were used. For each primer pair, PCR efficiency (E) and error were calculated using a standard curve derived from a pooled cDNA mixture serially diluted 4-fold over five measurement points. Each assay was performed in duplicate. The amplicon specificity was verified by 3% agarose gel electrophoresis. The quantification cycle (Cq) was automatically determined for each reaction by the LightCycler^®^ 480 SW 1.5 software using default parameters and the second derivative maximum method. The 2^−ΔΔCt^ method of Schmittgen and Livak [23] was used to calculate the relative expression levels.

### 2.5. Statistical Analyses

The data were collected from text files and Excel spreadsheets. Descriptive statistics, including mean values and standard deviations for each variable and time point, were calculated using Python 3.10.0 (Python Software Foundation, Wilmington, DE, USA) and R 4.3.1 (R Core Team, Vienna, Austria). The normality of data distribution was determined using the Shapiro–Wilk test, and the homogeneity of variances across groups was assessed using Levene’s test. Non-parametric tests were chosen for further analysis due to the sample size and deviation from normal distribution assumptions. Intergroup comparisons for each day of the experiment were conducted using the Mann–Whitney U test. Intra- and intergroup effects were evaluated using Repeated Measures ANOVA, with Mauchly’s test for sphericity and the Greenhouse–Geisser correction applied when necessary. The correlation between gene expressions was assessed using Spearman’s method. Matplotlib, a standard tool for data visualization, was used to create line and bar graphs. All analyses were conducted at a significant level of *p* < 0.05.

## 3. Results

### 3.1. Biochemical Parameters

**Figure 2:**
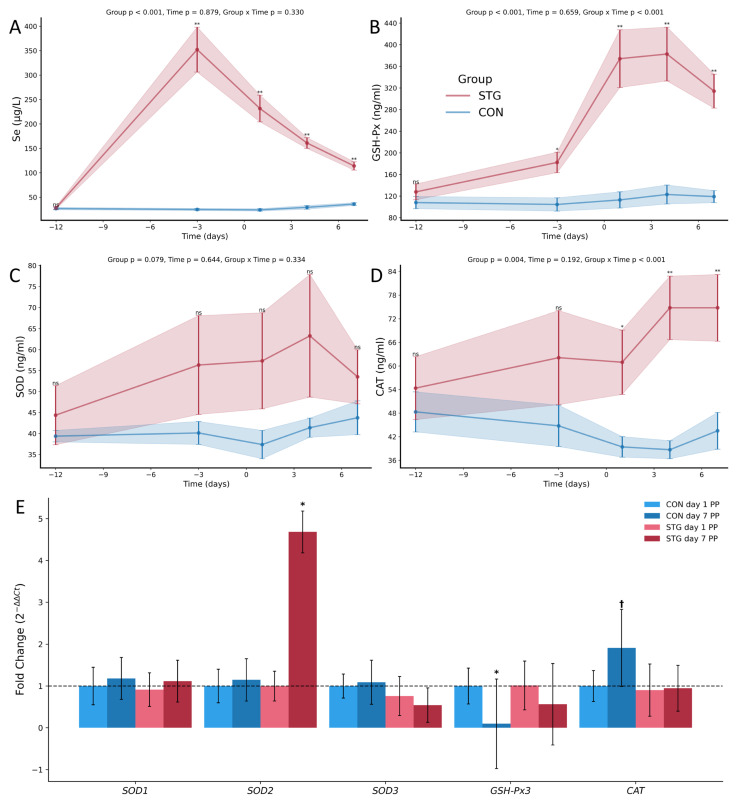
Depicts the concentration levels of serum components—panel (**A**): selenium (Se), panel (**B**): glutathione peroxidase (GSH-Px), panel (**C**): superoxide dismutase (SOD), and panel (**D**): catalase (CAT)—in cows on the 12th and 3rd day prepartum, as well as on the 1st, 4th, and 7th day postpartum. These concentrations are compared between the groups supplemented (STG) and not supplemented (CON) with selenitetriglycerides. Mean values with standard deviations (SD) are shown for each measurement. In addition, part (**E**) of the figure presents the fold changes in gene expression of antioxidant enzymes between the CON and STG groups. The *x*-axis categorizes the genes SOD1, SOD2, SOD3, GSH-Px3, and CAT, with separate bars for CON and STG samples on days 1 and 7 postpartum (PP). The y-axis measures the fold change in gene expression, scaled to display the entire range of observed data. The quantitative analysis of gene expression was conducted using RT-qPCR and the 2^−ΔΔCt^ method, with the fold change being represented as bars along with their associated standard error (SE). Statistical significance is denoted on the chart using specific symbols based on *p*-value thresholds: >0.01 is marked by ns (panels (**A**–**D**)), <0.01 is marked by **, denoting a highly significant difference, <0.05 is represented by *, indicating a statistically significant difference, and <0.1 is symbolized by †, suggesting a trend toward significance.

The concentration of selenium in the serum of cows from the control group (CON) and the experimental group (STG) in the first collection was similar and amounted to 26.82 μg/L (SD 6.02) and 28.17 μg/L (SD 6.41), respectively. Throughout the entire experimental period, the concentration of this microelement in the control group remained at a similar level, but in the last collection (7th day postpartum), it increased slightly and amounted to 35.96 μg/L (SD 7.02). In the group of cows that received selenitetriglycerides, the Se concentration in the second collection significantly increased to 352.25 μg/L (SD 112.59) and was significantly higher compared with the group of cows without supplementation. During the experiment, the serum Se concentration in these animals decreased significantly, and on the 7th day postpartum, it reached a concentration of 114.03 μg/L (SD 20.81), still remaining significantly higher compared with the control group cows (Figure 2A).

When analyzing the average activity of AST and GGT, no significant differences in these parameters were observed in relation to their initial values between groups or during the experiment (Table 4).

An analysis of glucose, triglycerides, and cholesterol levels across both groups showed no significant differences during the experimental timeline. However, a noteworthy observation was the marked decrease in levels of triglycerides (*p* = 0.01) and cholesterol (*p* < 0.001) following parturition in both the CON and STG groups (Table 4).

The concentration of non-esterified fatty acids in the first three samples did not differ significantly between the control and experimental groups of animals. After parturition, in the group of cows that received selenitetriglycerides, after a slight increase in the third collection, a decrease in NEFA concentration was observed in two subsequent collections. In the group of cows without supplementation, there was an increase in the concentration of this parameter after parturition. These differences were significant (*p* < 0.05) (Table 4). When analyzing the BHB concentration during the experiment, an increasing tendency for this indicator was observed in both groups of cows. There were no significant changes between the groups, except for the second collection (3 days before parturition), where in cows from the STG group there was a significant increase in this parameter compared with the control group (Table 4).

During the experiment, the concentration of albumin did not differ significantly between cows from the STG and CON groups. However, a decreasing trend in ALB concentration after parturition was observed in both groups. The protein concentration in the serum of cows that received selenitetriglycerides was significantly higher (*p* < 0.05) on the 3rd day before parturition and on the 4th and 7th days after parturition compared with the control group. As in the case of albumins, here too there was a decrease in the concentration of total protein after parturition in the serum of both groups of cows (Table 4).

### 3.2. Antioxidant Parameters

In the initial phase of the study, the concentration of glutathione peroxidase (GSH-Px) in the blood of the cows was found to be similar between the control (CON) group and the experimental (STG) group, with values of 86.18 ng/mL (SD 14.89) and 82.48 ng/mL (SD 24.29), respectively. In the group of cows without supplementation, the concentration of this enzyme decreased slightly before parturition to 68.05 ng/mL (SD 14.97) and then increased, and on the last day of the experiment, it amounted to 82.22 ng/mL (SD 12.02) (Figure 2). In cows that received selenitetriglycerides, there was a significant increase in GSH-Px concentration during the experiment compared with the first sampling date and compared with the group of cows without supplementation. The highest GSH-Px concentration was observed on the 4th day postpartum—178.97 ng/mL (SD 36.88). On the last day of the experiment, the concentration of this enzyme decreased slightly and amounted to 176.18 ng/mL (SD 28.50) (Figure 2B).

The concentration of superoxide dismutase on the first day of the experiment in the control and experimental groups was at a similar level (CON—39.38 ng/mL, SD 3.36, STG—44.37 ng/mL, SD 17.23). No significant differences in SOD concentration were observed between groups during the experiment, but a clear increase in SOD concentration was observed in the group of cows receiving selenitetriglycerides. The highest concentration of this enzyme in the STG group, similarly to the concentration of GSH-Px, was recorded on the 4th day postpartum—63.28 ng/mL (SD 35.71) (Figure 2C).

The analysis of catalase concentration in the first sampling revealed no significant differences between the CON and STG cow groups. During the experiment, in the group without supplementation, there was a non-significant decrease in CAT concentration immediately after parturition in the 3rd and 4th collections (Figure 2). In the STG group, there was a significant increase in the concentration of this enzyme during the experiment. As in the case of the two previous antioxidant enzymes in this group of cows, the highest CAT concentration was observed on the 4th day after parturition—74.76 ng/mL (SD 19.74) (*p* < 0.01) (Figure 2D).

### 3.3. Relative Gene Expression Analysis

The expression of superoxide dismutases (SOD1, SOD2, SOD3), GSH-Px3, and CAT was examined in the CON and STG groups. In the control group, SOD1 expression was at baseline (fold change = 1.00, SE = 0.45) and no significant changes were observed on the 7th day postpartum (fold change = 1.18, SE = 0.50, *p*-value = 0.70). SOD3 and CAT expressions were stable in the control groups and did not show statistically significant differences (Figure 2E). A significant increase in SOD2 expression was observed in the STG group (fold change = 4.68, SE = 0.50) with a *p*-value of 0.01, indicating a strong upregulation by selenium supplementation (Figure 2E). In addition, GSH-Px3 expression significantly decreased in the 7th day postpartum control group (fold change = 0.10, SE = 1.07, *p*-value = 0.04), while selenium supplementation reduced GSH-Px3 suppression, but the difference was not statistically significant (*p*-value > 0.05) (Figure 2E).

## 4. Discussion

The literature data regarding the proper concentration of Se in the bodies of cattle are divergent. Pavlata et al. [24] consider values above 100 μg/L to be sufficient, while concentrations below 70 μg/L are considered to be deficient, according to the authors. According to Gong and Xiao [25], the sufficient concentration of Se in the body of cows ranges from 70 to 79 µg/L. In turn, Stowe and Herdt [26] consider 40–70 μg/L to be a marginal selenium concentration, while according to Gerloff [27], a concentration below 40 μg/L means a significant deficiency of this element. Based on the presented data, it should be stated that the selenium concentration in cows determined in the first sampling, which did not exceed 30 μg/L, was very low and indicated hyposelenemia occurring in these animals. Studies conducted in the Czech Republic [28], aimed at determining the Se level in cattle, showed that deficit (<70 μg/L) and marginal (70–100 μg/L) levels occurred in 42% of cows, 80% of calves, 100% of heifers, and 90% of bulls [28]. The areas of north-eastern Poland, where the experiment was carried out, have a particularly low selenium content in the soil [8]. Free radicals and the oxidative stress they generate in the body of cows, especially during late pregnancy and early lactation, significantly affect the health, efficiency, and quality of products derived from them. To prevent oxidative stress and limit the consequences of its destructive impact, it is advisable to provide cows with feed and supplements rich in antioxidants. The participation of selenium in the construction of important enzymatic proteins causes this element to play an essential role in antioxidant processes. Selenitetriglycerides, as an organic form of selenium, effectively and quickly increase the Se concentration in the bodies of cows. In our studies, in the second sampling in the STG group, i.e., 9 days after starting supplementation, the concentration of this element was 352.25 µg/L (SD 112.59). Other studies on ruminants, where selenitetriglycerides were used, confirm the very rapid absorption of this form of selenium from the gastrointestinal tract. In cows administered selenitetriglycerides at the same dose used in the experiment, a twofold increase in serum selenium concentration was observed 24 h after oral administration of this form [17]. In the case of camels, which received a comparable dose, this increase was even greater, and within 24 h the Se concentration in the serum of these animals increased from 40.18 µg/L (SD 11.29) to 198.79 µg/L (SD 29.51) [19]. In calves, which received a single oral dose of selenitetriglycerides on the second day of life at 0.5 and 1 mg/kg BW, the serum selenium concentration increased within 24 h from 63.4 µg/L (SD 1.84) to 184.22 µg/L (SD 13.73) and from 63.17 µg/L (SD 1.72) to 200.33 µg/L (SD 21.42), respectively. After calving, the Se concentration in the STG group of cows decreased but still remained at a level considered sufficient [24,25].

Damage to hepatocytes in cows may be indicated by an increase in the activity of enzymes such as aspartate aminotransferase or gamma-glutamyl transferase in the serum [29,30]. In the studies conducted, no significant differences in AST and GGT activity were observed between the groups or in cows before and after calving. The lack of significant differences in the average activity of liver enzymes before and after calving indicates the proper functioning of the liver. Similarly, Sobiech et al. [31] showed no changes in the activity of these enzymes in cows that received selenium and vitamin E injections before calving, compared with cows without such supplementation. Changes in AST and GGT activity after supplementation with selenitetriglycerides were also not observed in cows and camels [18,19]. However, Li et al. [32] described an increase in aspartate aminotransferase activity in cows that added selenized yeast to their diet in various doses for three months. The authors did not observe a correlation between the increase in AST activity and the dose of supplemented selenized yeast.

No significant differences in glucose concentration were observed between the STG and CON groups of cows, which may suggest the lack of effect of Se supplementation on glucose metabolism in cows immediately after calving. Similarly, Gong and Xiao [33] showed no effect of prepartum Se-yeast use on glucose changes up to a week after calving. An increase in glucose concentration in the group of cows supplemented with Se before calving, compared with cows without supplementation, was observed by the authors only three weeks after calving. According to the authors of the above studies, the increase in glucose most likely occurred through increased gluconeogenesis efficiency in the liver.

In our study, serum triglyceride and cholesterol concentrations were not significantly different between STG and CON cows. These results confirmed the study by Khalili et al. [34], who observed no significant changes in triglyceride and cholesterol concentrations between cows supplemented with different forms of selenium compared with cows without supplementation. Similarly, Sobiech et al. [31] showed no effect of selenium and vitamin E use in cows before parturition on changes in concentrations of these parameters after parturition. In a study conducted in Xinjiang Brown cattle, there was no effect of selenized yeast supplementation at different doses on serum triglyceride concentrations [32]. However, the authors observed that animals supplemented with selenium at 0.3 mg/kg dry matter (DM) and 0.6 mg/kg DM for three months had a significant increase in serum cholesterol concentrations compared with the unsupplemented group, while animals receiving higher doses of Se (0.9 mg/kg DM and 1.2 mg/kg DM) had a significant decrease in this parameter. In our study, there was a decrease in triglyceride and cholesterol concentrations in cows just before and after parturition. Similar observations have been made by other authors [30,31]. The reduced mean value of triglyceride concentration in the postpartum period compared with the prepartum period may be explained by the uptake of triglycerides by the mammary gland for milk fat synthesis during lactation [30]. In addition, increased hepatic lipogenesis and ketogenesis contribute to a decrease in blood TG concentrations [35]. The reduction in cholesterol levels seen in this study is probably related to the increased need for steroid hormone synthesis in the endocrine glands of the mother and fetus.

Lipid mobilization is a typical response of cows to a negative energy balance in the periparturient period. Non-esterified fatty acids enter the bloodstream and thus provide a source of energy. The cow’s body is able to fully metabolize the released NEFA, but only in the amount that is necessary for energy needs related to gluconeogenesis [36]. However, when the amount of released non-esterified fatty acids exceeds the capacity of hepatocytes to metabolize them, triglycerides accumulate in the liver, disrupting its function and leading to the development of a fatty liver. Increased production of NEFA increases the production of reactive oxygen species during the β-oxidation process [37]. Oxidative stress may deepen the lipolysis process and consequently cause higher levels of non-esterified fatty acids in cows in the periparturient period, creating a vicious circle of lipolysis and ROS production [38]. High concentrations of NEFA and ROS production are part of metabolic stress and increase the risk of transitional period diseases, such as mastitis, retained placenta, ketosis, or fatty liver [36,38]. In our studies, an increase in free fatty acids in the serum was observed in cows without selenitetriglycerides supplementation after calving, while in the group of cows receiving selenitetriglycerides, the concentration of these compounds decreased in the last two collections. The results of our studies suggest that the use of selenitetriglycerides before calving affects the relief of oxidative stress in the first week after calving. This is confirmed by studies conducted on cows that were fed Se-yeast in their feed for 4 weeks before calving [33]. According to the authors, administering Se before calving may contribute to an increase in insulin levels after calving. Insulin, in turn, is responsible for the synthesis of triglycerides in adipose tissue and has an antilipolytic effect, which in turn reduces the concentration of NEFA in the blood of cows after calving. The strong antioxidant properties of selenium derived from selenitetriglycerides and the associated reduction in NEFA in the blood are confirmed by other studies conducted on cows [17]. Also, research by Ren et al. [39] showed that Se enhances the antioxidant effect of the body, which results in a reduced incidence of metabolic diseases in dairy cows in the periparturient period. Moreover, they documented that selenium supplementation affects the reduction of alpha S1 casein, apolipoprotein A-I, and apolipoprotein C-II expression and up-regulates macrophage-stimulating protein and chromogranin-A in cows in the periparturient period. Reducing alpha-S1 casein, apolipoprotein A-I, and apolipoprotein C-II reduces lipid activity, thus controlling excessive fat mobilization and reducing the risk of ketosis and hepatic steatosis [39]. However, Hall et al. [40] and Sobiech et al. [31] did not observe the effect of administering Se before calving on changes in NEFA concentration in the serum after calving.

In our study, there was no effect of selenitetriglycerides supplementation on serum BHB concentrations. This is confirmed by studies by other authors [17,31,33]. The increase in BHB concentration observed in our study is associated with the increased energy requirements of cows during the transition period. A significant increase in BHB is considered an indicator of excessive NEB in dairy cows [41]. Ospina et al. (2010) report that serum NEFA concentrations above 0.6 mmol/L and BHB concentrations above 1.0 mmol/L before parturition or in early lactation are associated with an increased risk of tract displacement, clinical ketosis, uterine inflammation, or placental retention within the first 30 days of lactation. In our own study, both NEFA and BHB concentrations in cows of both groups did not exceed the above levels during the course of the experiment.

No significant differences in albumin concentration in serum were observed between the groups of cows during the experiment. No changes in the serum albumin concentration of Xinjiang brown cattle after administration of selenized yeast with different Se content to the diet were also observed by Li et al. [32]. However, Gong and Xiao [33] showed higher albumin concentrations on the 7th and 21st days after calving in cows that were supplemented with Se-yeast before calving compared with cows without supplementation. Albumin is a negative acute phase protein and is used to detect inflammation in the body. Low albumin levels in the blood of cows may also indicate liver dysfunction. According to Bertoni et al. [42], in cows with liver dysfunction after calving, the albumin concentration is less than 30 g/L. In our studies, the concentration of this indicator was above these values in both groups throughout the duration of the experiment. Considering other parameters used to assess liver activity (AST, GGT), it can be concluded that during the studied period after calving, there was no disturbance in hepatocyte function. It should be emphasized that albumin takes part in many biochemical processes occurring in the body, including antioxidant defense. This protein can bind almost all known endogenous compounds, metal ions, and xenobiotics and has several enzymatic activities: pseudo-esterase, paraoxonase, phosphotriesterase, thioesterase, glutathione peroxidase, and cysteine peroxidase. The participation of albumin in redox reactions is non-specific, due to its very high concentration in the extracellular space and relatively quick renewal [43]. Oxidative stress can be measured using biological markers, which include potential antioxidant capacity (PAO). This parameter determines the total antioxidant capacity in the serum using the copper ion reduction reaction [44]. Potential antioxidant capacity has been shown to be positively correlated with albumin value in cows with postpartum diseases such as ketosis and abomasum displacement [45].

Total protein concentration in the blood is one of the indicators of nitrogen metabolism in the body; it undergoes constant dynamic changes and depends on the amount of protein and energy in the feed, the age of the cow, the stage of lactation, and the season [46]. In our studies, a significantly higher concentration of total protein in serum was observed in cows from the STG group. The effect of administering organic and inorganic forms of selenium before calving on increasing the concentration of total protein in serum after calving was also demonstrated by Khalili et al. [34]. Many studies have shown a decrease in total protein concentration in the serum of cows before calving and in early lactation [47,48]. The decrease in TP concentration in the blood of cows after calving, which was also observed in our studies, may result from the transfer of blood albumins and globulins into the udder [45], but also from the use of protein reserves to compensate for the energy deficit [31,49]. Free radicals can cause oxidative damage to plasma proteins, resulting in the formation of advanced oxidation protein products (AOPP) [50]. Kolagal et al. [51] showed a decrease in total protein and albumin concentrations and an increase in AOPP in patients who developed oxidative stress associated with uremia. The higher concentration of total protein in cows from the STG group may indicate the strong protective properties of selenitetriglycerides against the oxidation of blood proteins in cows in the periparturient period.

Enzymatic defense systems, including superoxide dismutase, catalase, glutathione peroxidase, and others, protect DNA and mitochondria from damage caused by oxidative stress. In the present study, cows in the selenitetriglycerides-supplemented group had a significant increase in GSH-Px concentration compared with the unsupplemented group. Peroxidase activity depends on the selenium content of the diet and is positively correlated with selenium intake [13,52]. The effect on the increase of glutathione peroxidase in the animal body after selenitetriglyceride supplementation was confirmed in calves [19] and pigs [20]. In contrast, Zagrodzki et al. [53] describe the lack of effect of selenitetriglycerides administration to sheep on increasing the activity of selenoenzymes (glutathione peroxidase, iodothyronine deiodinases, and thioredoxin reductase) in the brain, adrenal glands, kidneys, liver, and thyroid. The increase in glutathione peroxidase concentration in the STG group occurred at the second dose (9 days after starting supplementation), and the enzyme reached its highest concentration at the fourth dose (4 days postpartum). In calves given a single dose of selenitetriglycerides, an increase in GSH-Px activity was observed on day 5 after selenium administration at a dose of 0.5 mg/kg BW and on day 10 after selenium administration at a dose of 1 mg/kg BW [19]. In contrast, cows given selenium by sodium selenate injection before parturition showed an increase in glutathione peroxidase activity after 10 days [31]. The difference in time between Se administration and GSH-Px increase may be related to the fact that selenium is first used to replenish deficiencies in priority body structures (brain, endocrine glands, reproductive organs) and only later transported to the liver, heart, skeletal muscle, and erythrocytes [54] and the processes of Se incorporation into erythrocytes during erythropoiesis and GSH-Px biosynthesis in the blood cell [55]. In the control group, glutathione peroxidase levels were significantly low throughout the experiment. The decrease of GSH-Px in the blood of cows during the dry period and the first days of lactation is confirmed by other studies [31,56,57]. Pilarczyk et al. [56] found the highest GSH-Px activity in cow serum during the first stage of lactation, which the authors suggest may indicate that the production of ROS and their derivatives is highest during this period, exposing cows to significant oxidative stress.

In our study, a non-significant increase in superoxide dismutase and a significant increase in catalase were observed in the STG group. The effect on the increase of SOD and CAT activity in cows given Se-yeast prepartum is confirmed by Gong and Xiao [58]. As mentioned in the introduction, in addition to peroxidase, selenium regulates many other genes responsible for mitigating the effects of oxidative stress, including SOD and CAT. Reducing dietary levels of selenium and other antioxidants results in a decrease in SOD activity. It has been suggested that this may be influenced by GSH-Px4, which is most likely involved in SOD activation [14]. In the CON group, SOD concentrations did not change, while a non-significant decrease in this enzyme was observed for CAT over the course of the experiment. The lack of change in SOD activity during the transition period has also been shown in other studies [58,59,60]. A decrease in catalase in the peri- and postpartum period has been described by other authors [58,61], suggesting that the enzymatic antioxidant system in peripartum dairy cows is insufficient to counteract the development of oxidative stress in early lactation.

Superoxide dismutases (SODs) are the first and most important line of enzymatic defense systems against reactive oxygen species, in particular against superoxide anion radicals [62]. So far, three different SOD isoforms have been identified in mammals: SOD1 (CuZn-SOD), SOD2 (Mn-SOD), and SOD3 (EC-SOD). In our study, no changes in SOD1 and SOD3 expression were observed between groups, which may indicate that Se supplementation has no effect on the gene expression of these enzymes in the liver. Tsuchiya et al. [63] showed significantly lower SOD3 gene expression in cows at 2 weeks postpartum compared with 6 weeks postpartum. Although SOD2 is expressed at high levels in many cell types and tissues, its expression is highly regulated by a variety of intracellular and environmental signals [64]. Our study showed a significant increase in the expression of the gene encoding SOD2 in the liver of cows after Se supplementation on day 7 postpartum. The effect of selenium supplementation in rats on increasing SOD2 levels in the liver, which was due to an increase in SOD2 transcription in hepatocytes, was also described by Shilo et al. [65], confirming the effect of selenium on improving antioxidant defenses in hepatocytes. The authors emphasize that mild oxidative stress induced by Se, which is both an antioxidant and a prooxidant, may indirectly lead to increased SOD2 expression. Such oxidative stress may contribute to the development of a mild, subclinical inflammatory response and thus increase SOD2 levels. Selenium can therefore act as a preparative measure against stronger, more damaging stresses, such as bacterial lipopolysaccharide (LPS).

Catalase is an enzyme that has functions both in the catabolism of H_2_O_2_ and in the oxidation of exogenous substrates such as methanol or ethanol. Our study showed no significant changes in CAT expression between groups or between sampling dates. Tsuchiya et al. [63] also did not observe changes in CAT expression in hepatocytes after parturition. Furthermore, the authors showed no significant differences in the expression of this antioxidant in hepatocytes between healthy cows and cows with subacute ruminal acidosis (SARA). In a study in broilers, an increase in dietary selenium and vitamin E levels was associated with an increase in the expression of CAT, SOD, and GSH-Px in liver tissue [66]. The authors highlight the fact that vitamin E and/or Se may not only act as exogenous antioxidants but also as gene regulators, controlling the expression of endogenous antioxidant enzymes. In the above experiment, broilers were given vitamin E and/or Se for a period of 30 days, were healthy, and were not exposed to any stress factors. In our study, no effect of Se supplementation was observed on the increase of CAT expression in the liver, which may be related both to the short period of Se supplementation in the form of selenitetriglycerides (12 days) and the oxidative stress that occurred in cows during the peripartum period, but also to the difference in intracellular metabolism that occurs in ruminants.

In our study, unsupplemented cows with serum selenium concentrations well below reference levels showed a significant decrease in liver GSH-Px3 expression on day 7 postpartum compared with the first sampling date. No significant changes in GSH-Px3 expression were observed in hepatocytes from cows in the STG group between days 1 and 7 postpartum. Zhang et al. [67] conducted a study on mice supplemented with selenium at different doses: 0.045 mg Se/kg (Se-deficient group), 0.1 mg Se/kg (Se-adequate group), 0.4 mg Se/kg (Se-supernutrition group), and 0.8 mg Se/kg (Se-excess group). Analysis by RT-qPCR showed that mRNA levels of GSH-Px1, GSH-Px3, and GSH-Px4 were reduced in the livers of mice with the lowest (0.045 mg/kg) and highest (0.8 mg/kg) dietary Se concentrations. In animals receiving selenium at a 0.4 mg/kg diet, hepatic GSH-Px3 mRNA levels were reduced compared with Se-adequate mice (0.1 mg/kg), with unchanged mRNA levels for GSH-Px1 and GSH-Px4, suggesting that hepatic GSH-Px3 is more sensitive to Se overexposure than other genes, according to the authors. The results presented here correspond to those obtained in our own research and confirm that the dose of selenium applied in the form of selenitetriglycerides (0.5 mg Se/kg BW) does not adversely affect hepatocytes and, at the same time, effectively increases selenium levels and antioxidant capacity in the body of cows during the periparturient period. Studies in cows supplemented with nano-selenium significantly increased mRNA expression of GSH-Px1, GSH-Px2, GSH-Px4, TXNRD2, and TXNRD3 in the mammary gland compared with cows without supplementation [68]. In contrast, supplementation with this form of Se had no effect on the mRNA expression of the GSH-Px3 and TXNRD1 genes.

## 5. Conclusions

The investigation into the effects of supplementation with selenitetriglycerides in Holstein-Friesian cows during the peripartum period has yielded significant and noteworthy discoveries that enhance our comprehension of the role of selenium in the management of oxidative stress in dairy cattle. One of the crucial outcomes of this study is the successful enhancement of selenium levels in cows through the administration of selenitetriglycerides, as evidenced by a pronounced increase in serum selenium levels in the group that received the supplementation. It is important to highlight that the study also revealed a specific upregulation of the SOD2 gene, thus emphasizing the targeted action of selenium on the mitochondrial antioxidative defense mechanisms. Conversely, the expressions of SOD1, SOD3, and CAT remained unaltered, indicating a selective influence of selenium on antioxidative genes. Furthermore, the study also observed stable levels of metabolic parameters such as glucose, triglycerides, and cholesterol, and a reduction in these parameters was observed postpartum in both the control and supplemented groups. Notably, the activities of liver enzymes and the levels of serum proteins did not exhibit any significant changes, suggesting the maintenance of liver health and indicating a potential protective effect of selenium against oxidation. In addition, the decrease in non-esterified fatty acids post-calving in the group that received the supplementation suggests an alleviation of oxidative stress and an improvement in metabolic health, possibly due to the antioxidative properties possessed by selenium. Taking all these findings into consideration, it is evident that this research underscores the potential of selenium supplementation, particularly in the form of selenitetriglycerides, to enhance the antioxidant status and support the health of dairy cows during the critical peripartum period. These insights have practical implications for dietary management in the domain of dairy farming, with the primary goal of reducing oxidative stress and enhancing both animal welfare and productivity.

## Figures and Tables

**Table 1:** Composition of the Prepartum Diet for Dry-Off Cows.

Ingredient	Weight/Cow	DM ^1^	Final DMI ^1^
	(kg)	(%)	(kg)
Corn Silage	17.00	31.00	5.27
Grass Straw	11.00	23.00	2.53
Straw	1.00	84.00	0.84
Soybean Meal	1.80	88.00	1.58
Rapeseed Meal	1.30	88.00	1.14
Vitamin-mineral preparation	0.40	88.00	0.35
Total	32.50		11.72

^1^ Dry matter intake (DMI) is calculated by multiplying the dry matter percentage (DM%) of the feed by the weight (kg) of the feed offered. The target daily DMI is set at 2% of a cow’s body weight. The vitamin-mineral preparation contains: Vitamin A, 225,000 IU/kg; Vitamin D3, 60,000 IU/kg; Vitamin E, 2000 mg/kg; Vitamin B1, 15 mg/kg; Vitamin B2, 22 mg/kg; Niacin, 100 mg/kg; Pantothenic acid, 90 mg/kg; Vitamin B6, 12 mg/kg; Folic acid, 20 mg/kg; Vitamin B12, 450 mcg/kg; Biotin, 10,000 mcg/kg; β-Carotene, 300 mg/kg; Iron (as ferrous sulfate), 380 mg/kg; Manganese (as manganous oxide), 1400 mg/kg; Manganese (as manganese chelate), 280 mg/kg; Zinc (as zinc oxide), 1750 mg/kg; Zinc (as zinc chelate), 650 mg/kg; Copper (as copper sulfate pentahydrate), 500 mg/kg; Copper (as copper chelate), 130 mg/kg; Iodine (as calcium iodate), 42 mg/kg; Selenium (as sodium selenite), 8.2 mg/kg; Selenium (as selenomethionine), 5.5 mg/kg; Cobalt (as cobalt carbonate), 22.5 mg/kg; Citric acid, 10 mg/kg; Propyl gallate, 20 mg/kg; B.H.T. (E321), 20 mg/kg; Clinoptilolite (of sedimentary origin), 50,000 mg/kg.

**Table 2:** Nutritional Components in Early Lactation Cow Feeding.

Ingredient	Weight/Cow	DM ^1^	Final DMI ^1^
	(kg)	(%)	(kg)
Corn Silage	29.00	31.00	8.99
High Starch Concentrate	3.00	88.00	2.64
Concentrate	3.00	88.00	2.64
Alfalfa Silage	10.00	41.00	4.10
Haylage	2.50	47.00	1.18
Straw	0.30	84.00	0.25
Beet Molasses	0.50	75.00	0.38
Brewer’s spent grain	5.00	21.00	1.05
Soybean Meal	1.80	88.00	1.58
Rapeseed Meal	1.30	88.00	1.14
Sodium Bicarbonate	0.25	99.00	0.25
Propylene Glycol	0.13	95.00	0.12
Rumen-Protected Fat	0.20	97.00	0.19
Vitamin-mineral preparation	0.66	88.00	0.58
Total	57.64		25.10

^1^ Dry matter intake (DMI) is calculated by multiplying the dry matter percentage (DM%) of the feed by the weight (kg) of the feed offered. The target daily DMI is set at 2% of a cow’s body weight. The vitamin-mineral preparation contains: Vitamin A, 225,000 IU/kg; Vitamin D3, 60,000 IU/kg; Vitamin E, 2000 mg/kg; Vitamin B1, 15 mg/kg; Vitamin B2, 22 mg/kg; Niacin, 100 mg/kg; Pantothenic acid, 90 mg/kg; Vitamin B6, 12 mg/kg; Folic acid, 20 mg/kg; Vitamin B12, 450 mcg/kg; Biotin, 10,000 mcg/kg; β-Carotene, 300 mg/kg; Iron (as ferrous sulfate), 380 mg/kg; Manganese (as manganous oxide), 1400 mg/kg; Manganese (as manganese chelate), 280 mg/kg; Zinc (as zinc oxide), 1750 mg/kg; Zinc (as zinc chelate), 650 mg/kg; Copper (as copper sulfate pentahydrate), 500 mg/kg; Copper (as copper chelate), 130 mg/kg; Iodine (as calcium iodate), 42 mg/kg; Selenium (as sodium selenite), 8.2 mg/kg; Selenium (as selenomethionine), 5.5 mg/kg; Cobalt (as cobalt carbonate), 22.5 mg/kg; Citric acid, 10 mg/kg; Propyl gallate, 20 mg/kg; B.H.T. (E321), 20 mg/kg; Clinoptilolite (of sedimentary origin), 50,000 mg/kg.

**Table 3:** The quantitative reverse transcription-PCR (qRT-PCR) primer sequences applied in the study and the amplicons’ details.

Gene Symbol	Primer Sequences	Annealing Temp.	T_m_	Amplicon Size	E	Error
	(F—Forward/R—Reverse) (5′-3′)	(°C)	(°C)	bp	10^−1/slope^	MSE
SOD1	F—TGTTGCCATTCGTGGATATTGTAG R—CCCAAGTCATCTGGTTTTTCATG a	60	80.2	103	1.98	0.012
SOD2	F—CGCTGGAGAAGGGTGATGTT R—GATTTGTCCAGAAGATGCTGTGAT	60	82.5	99	1.99	0.006
SOD3	F—GCAGCAGATGGGCTCCAA R—GCATCATCTCCTGCCAGATCTC	58	85.3	80	2.06	0.011
GSH-Px3	F—GTCAACGTGGCCAGCTACTGA R—CAGAATGACCAGACCAAATGGTT	60	83.9	93	1.99	0.019
CAT	F—GGAAACGCCTGTGTGAGAAC R—CTGCGTTCTTAGGTTTCTCCTC	58	81.7	159	1.95	0.005
GAPDH reference gene	F—GTCTTCACTACCATGGAGAAGG R—TCATGGATGACCTTGGCCAG	60	86.1	197	2.03	0.011
RPL32 reference gene	F—AAAGAGGACCAAGAAGTTCATTAG R—CGCCAGTTCCGCTTGATTT	60	78.1	66	1.98	0.013

E—PCR efficiency calculated using formula E = 10^−1/slope^, Error value—mean squared error of the single data point fit to the regression line, T_m_—melting temperature curve peak analysis.

**Table 4:** Biochemical parameters in cows supplemented (STG) and not supplemented (CON) with selenitetriglycerides on the 12th and 3rd days prepartum and on the 1st, 4th, and 7th days postpartum (mean ± SD). Different superscripts (a and b) indicate a significance level of *p* < 0.05.

		Day Relative to Parturition				
	Group	−12	−3	1	4	7
		(Mean ± SD)	(Mean ± SD)	(Mean ± SD)	(Mean ± SD)	(Mean ± SD)
AST (U/L)	CON	75.00 ± 12.40	70.00 ± 6.90	82.67 ± 13.95	84.16 ± 9.31	78.50 ± 14.80
	STG	66.33 ± 10.76	68.83 ± 7.65	85.33 ± 13.39	87.83 ± 22.15	88.17 ± 16.75
GGT (U/L)	CON	28.17 ± 2.48	27.33 ± 3.83	31.67 ± 8.36	28.33 ± 4.41	28.16 ± 5.91
	STG	26.00 ± 2.53	26.17 ± 3.37	29.12 ± 10.00	27.83 ± 3.37	29.67 ± 3.44
GLU (mmol/L)	CON	3.26 ± 0.23	3.45 ± 0.40	3.63 ± 0.81	3.37 ± 0.50	3.59 ± 0.49
	STG	3.19 ± 0.39	4.13 ± 0.84	3.15 ± 0.65	3.26 ± 0.45	3.22 ± 0.43
TG (mmol/L)	CON	0.19 ± 0.09	0.22 ± 0.06	0.13 ± 0.09	0.09 ± 0.03	0.11 ± 0.05
	STG	0.15 ± 0.10	0.19 ± 0.13	0.10 ± 0.04	0.07 ± 0.03	0.08 ± 0.05
CHOL (mmol/L)	CON	2.92 ± 0.70	2.83 ± 0.67	2.37 ± 0.37	2.27 ± 0.50	2.36 ± 0.52
	STG	2.55 ± 0.58	2.71 ± 0.68	2.19 ± 0.50	2.05 ± 0.26	2.14 ± 0.33
NEFA (mmol/L)	CON	0.44 ± 0.22	0.41 ± 0.18	0.56 ± 0.30	0.55 ± 0.31 ^a^	0.58 ± 0.27 ^a^
	STG	0.39 ± 0.12	0.39 ± 0.15	0.51 ± 0.15	0.41 ± 0.08 ^b^	0.36 ± 0.17 ^b^
BHB (mmol/L)	CON	0.69 ± 0.14	0.66 ± 0.13 ^a^	0.64 ± 0.16	0.70 ± 0.11	0.72 ± 0.23
	STG	0.61 ± 0.20	0.85 ± 0.15 ^b^	0.80 ± 0.14	0.83 ± 0.15	0.82 ± 0.20
ALB (g/L)	CON	39.70 ± 1.91	39.13 ± 0.95	38.46 ± 1.38	37.40 ± 1.75	38.18 ± 2.66
	STG	39.26 ± 2.41	38.96 ± 1.62	38.21 ± 3.84	36.65 ± 3.56	36.91 ± 3.49
TP (g/L)	CON	68.88 ± 2.95	67.22 ± 3.76 ^a^	66.27 ± 3.59	63.70 ± 4.33 ^a^	64.25 ± 3.56 ^a^
	STG	72.68 ± 5.35	72.95 ± 4.22 ^b^	68.53 ± 2.37	67.87 ± 3.28 ^b^	68.37 ± 2.31 ^b^

## Data Availability

None of the data were deposited in an official repository. All the data obtained in the present research are presented in this manuscript. The data that support the study findings are available from the authors upon request.
